# Management of uncomplicated malaria among children under five years at public and private sector facilities in Mali

**DOI:** 10.1186/s12889-020-09873-1

**Published:** 2020-12-09

**Authors:** Seydou Fomba, Diakalia Koné, Bakary Doumbia, Diadier Diallo, Thomas Druetz, Lia Florey, Thomas P. Eisele, Erin Eckert, Jules Mihigo, Ruth A. Ashton

**Affiliations:** 1 Programme National de Lutte contre le Paludisme, Bamako, Mali; 2 Info-Stat, Bamako, Mali; 3 ICF, Bamako, Mali; 4 Center for Applied Malaria Research and Evaluation, Tulane School of Public Health and Tropical Medicine, New Orleans, LA USA; 5 Department of Social and Preventive Medicine, School of Public Health, University of Montreal, Montreal, QC Canada; 6 President’s Malaria Initiative, United States Agency for International Development, Washington, DC USA; 7 RTI International, Washington, DC USA; 8 President’s Malaria Initiative, United States Agency for International Development, Bamako, Mali; 9 MEASURE Evaluation, Center for Applied Malaria Research and Evaluation, Tulane School of Public Health and Tropical Medicine, New Orleans, LA USA

**Keywords:** Mali, Malaria, Case management, Febrile illness, Children under five years

## Abstract

**Background:**

Prompt and effective malaria diagnosis and treatment is a cornerstone of malaria control. Case management guidelines recommend confirmatory testing of suspected malaria cases, then prescription of specific drugs for uncomplicated malaria and for severe malaria. This study aims to describe case management practices for children aged 1–59 months seeking treatment with current or recent fever from public and private, rural and urban health providers in Mali.

**Methods:**

Data were collected at sites in Sikasso Region and Bamako. Health workers recorded key information from the consultation including malaria diagnostic testing and result, their final diagnosis, and all drugs prescribed. Children with signs of severe diseases were ineligible. Consultations were not independently observed. Appropriate case management was defined as both 1) tested for malaria using rapid diagnostic test or microscopy, and 2) receiving artemisinin combination therapy (ACT) and no other antimalarials if test-positive, or receiving no antimalarials if test-negative.

**Results:**

Of 1602 participating children, 23.7% were appropriately managed, ranging from 5.3% at public rural facilities to 48.4% at community health worker sites. The most common reason for ‘inappropriate’ management was lack of malaria diagnostic testing (50.4% of children). Among children with confirmed malaria, 50.8% received a non-ACT antimalarial (commonly artesunate injection or artemether), either alone or in combination with ACT. Of 215 test-negative children, 44.2% received an antimalarial drug, most commonly ACT. Prescription of multiple drugs was common: 21.7% of all children received more than one type of antimalarial, while 51.9% received an antibiotic and antimalarial. Inappropriate case management increased in children with increasing axillary temperatures and those seeking care over weekends.

**Conclusions:**

Multiple limitations in management of febrile children under five were identified, including inconsistent use of confirmatory testing and apparent use of severe malaria drugs for uncomplicated malaria. While we cannot confirm the reasons for these shortcomings, there is a need to address the high use of non-ACT antimalarials in this context; to minimize potential for drug resistance, reduce unnecessary expense, and preserve life-saving treatment for severe malaria cases. These findings highlight the challenge of managing febrile illness in young children in a high transmission setting.

**Supplementary Information:**

The online version contains supplementary material available at 10.1186/s12889-020-09873-1.

## Background

Access to prompt and effective malaria diagnosis and treatment is one of the foundations of malaria prevention and control strategies [1]. While substantial progress has been made in reducing malaria burden since mass intervention scale-up in the early 2000s, progress has since stalled [2]. It is essential that National Malaria Programs review their performance regularly to assess if existing policies are appropriate, and that patients are receiving quality diagnosis and treatment of malaria [3]. Effective malaria case management is characterized by parasitological testing of all suspected malaria cases and prompt provision of quality-assured treatment, including identification and management of severe malaria [4]. Prompt and effective treatment of malaria is particularly important among children under 5 years; this age group accounted for 61% of global malaria deaths in 2017 [2]. Use of confirmatory diagnostics and ensuring appropriate dosing of quality-assured first-line antimalarials improves malaria treatment outcomes and reduces the risk of drug resistance. Improved algorithms for management of patients who test negative for malaria are also important in management of acute febrile illness more broadly [5].

Mali adopted a policy of artemisinin-based combination therapy (ACT) for treatment of uncomplicated malaria in 2005, replacing chloroquine [6]. Artemether-lumefantrine (AL) is the first-line ACT, with artesunate-amodiaquine (AA) the second-line. National treatment guidelines indicate that children with severe malaria should receive an injection of either artesunate, artemether, or quinine (according to availability), followed by a full dose of ACT as soon as they are able to safely take oral medication [7].

The public health system in Mali is divided into 65 functional health districts, with each health district comprising one referral health center (*Centres de Santé de Réference*, CSRef) and multiple community health centers (*Centres de Santé Communautaire*, CSCom). The health district is led by a medical chief, with a malaria focal person allocated to every health district. CSRefs comprise the first reference level of the health system, with a second reference level consisting of eight regional hospitals, and third reference level comprising five national reference hospitals. Health system governance is decentralized, with a strong emphasis on community participation to extend health service coverage [8]. There is a shortage of staff at all levels of the public health system, but particularly at facilities below national level [8]. A program of “medicalization” of CSComs began in 2011, aiming to appoint medical doctors at CSComs. In 2014, 100% of CSComs in Bamako were staffed by a medical doctor, but only 19–28% of CSComs in the regions of Kayes, Koulikoro, Segou and Sikasso [9]. CSComs operate on a cost recovery system based on the Bamako Initiative and are managed by community health associations, who are responsible for managing the CSCom staff and operations, paying salaries, purchasing diagnostic and treatment commodities, and collecting income from drug sales and user fees [8]. Community health workers (CHWs) have been introduced to increase access to basic health services in areas > 5 km from a CSCom, and report to CSComs. CHWs in Mali are supported by various donors including Global Fund, UNICEF and USAID, or by financial incentives from local political authorities. Diagnosis of malaria by rapid diagnostic test (RDT) is free for all, but microscopy diagnosis is free only for children under 5 years (U5) and pregnant women. Malaria treatment is free for U5s and pregnant women at all levels of the health system. Other members of the population must pay for malaria treatment, but the price is kept low to minimize financial barriers to treatment. A 2015 national malaria survey indicated that there were differences in treatment seeking between rural and urban areas: while 67% of children under 5 years with fever in an urban area were taken to a health provider, the equivalent value in rural areas was only 45% of febrile children [10].

Mali has implemented seasonal malaria chemoprevention (SMC) policies since 2012, reaching national scale in 2017. Children aged 3–59 months receive up to four rounds of sulfadoxine-pyrimethamine (SP) and amodiaquine (AQ) during the peak malaria season, at one-month intervals. Children with fever on the day of the SMC campaign are tested using an RDT, and those with a positive RDT receive the standard dose of ACT instead of SP and AQ.

Monitoring of malaria case management procedures allows malaria control programs to assess if diagnosis and treatment policies are being followed; particularly the use of confirmatory diagnostics, adherence to test results when prescribing treatment, and choice of appropriate drug and dosing for the patient. While household surveys such as the Malaria Indicator Survey collect information about caregivers’ report of care received by recently febrile (fever in the last 2 weeks) U5s, alternative tools such as health facility surveys and patient exit interviews give a more direct assessment of procedures applied by healthcare providers, and End User Verification surveys (EUV) combine information on commodity availability with review of patient records at facilities. Systems effectiveness approaches combine ACT efficacy, treatment-seeking, malaria testing rate, proportion of test-positives prescribed ACT, and patient comprehension of the ACT regimen [11, 12]. Other studies evaluating malaria case management have focused on procedures at the facility: assessment of fever or fever history, malaria testing, treatment prescribed and dose [13, 14].

Limitations in case management identified by previous studies included failure to perform diagnostic testing on all suspected malaria cases [13–15], prescription of antimalarial drugs for test negative patients [14, 16, 17], or ACT not being prescribed to patients with confirmed malaria [18, 19]. Additional shortcomings in other settings include ‘poly-pharmacy’ whereby multiple antimalarials are given at once, incorrect dose prescription [13, 20], and lack of referral/admission or pre-referral treatment for severe malaria [21]. Presence of substandard or degraded drugs at facilities also impacts case management effectiveness. Stock-out of key commodities, or ‘rationing’ in anticipation of future stock-outs also likely contribute to some of these shortcomings, in addition to the beliefs and preferences of health workers, patients, and caregivers [22, 23].

Data from supervision visits to health facilities in Mali suggest that adherence to negative test results is lower than adherence to positive results, suggesting lack of trust in test results by health workers [24]. Stock outs of RDTs are thought to be responsible for underperformance in confirmatory testing, but stock outs of ACT do not appear to be responsible for lack of adherence to positive test results, suggesting either deviation from the case management protocol or record-keeping problems [25]. EUV in Mali in 2017 found that although some facilities did not follow case management protocols, overall adherence by facilities was good, but 19% of facilities had experienced a stockout of RDTs in the previous month lasting more than 3 days, and 15% had experienced a stockout of one of the ACT doses used for U5s [26].

Consequences of deficiencies in case management can include increased progression to severe malaria if patients with malaria are not adequately identified and treated; wastage of drugs if prescribed to malaria-negative patients, increasing costs for the malaria control program and reducing supply of drugs for those in need; failure to clear infection or promotion of drug resistance if incorrect drugs or doses are prescribed; and failure to address other potential causes of fever such as typhoid fever, diarrhea and pneumonia.

This paper reports malaria case management practices recorded during a treatment recall validation study in a high transmission setting in Mali, including public health facilities in urban and rural areas, CHW sites, and private health facilities in urban areas [27]. The aim was to conduct secondary analysis of existing data collected from different types of health provider and locations for a treatment recall validation study to describe malaria testing and treatment practices among febrile U5s. Specifically, we report data on use of parasitological testing among U5s attending with complaint of fever, adherence to diagnostic test result by health workers, medicines prescribed to children in the study, and explore factors associated with deviation from malaria case management guidelines.

## Methods

### Study site

The study was conducted in urban areas of Sikasso Region and Bamako District, and in rural areas of Niena District in Sikasso Region. Sikasso Region experiences high malaria transmission typical of the Sudano-Guinean zone with a seasonal peak during June to November, while Bamako experiences lower levels of malaria transmission due to its urban environment and location in the Sahelian zone [28]. Four types of health facility were included in the study: two urban private facilities, two urban public facilities, two rural public facilities and nine CHWs. Full details of site selection are detailed elsewhere [27].

### Data collection procedures

The data were collected as part of a study aiming to validate caregivers’ recall of diagnosis and treatment procedures, and detailed procedures for participant identification, enrolment and data collection have been described elsewhere [27]. Briefly, any child aged 1–59 months (U5) attending the participating health facilities or CHWs with current or recent fever was eligible for inclusion. Children with signs of severe illness were ineligible. Immediately after the consultation, health workers recorded key information about the consultation using a short questionnaire: malaria diagnostic tests used, test result, final diagnosis made by the health worker, and all drugs prescribed to the patient (Supplementary File 1).

A study team member was located at participating health facilities and reviewed consultation questionnaires on an ongoing basis but did not directly observe consultations. Health workers were informed of the general aim of the study (to explore caregivers’ recall of drugs received) but advised to continue their consultations according to their normal practices. To avoid any bias regarding the disease of interest, caregivers of the participating U5s were told that the study was related to general issues of child health. Consent was requested from caregivers for inclusion of their child in the study. Caregivers who declined consent were not included in the study, and the study-specific consultation record for their U5 was destroyed. No information on health worker demographics, qualifications or duration of service at the health facility were recorded, and participants’ *Plasmodium* infection status or symptoms were not independently assessed by the survey team.

In order to assess any potential confusion regarding the administration of preventive medications for seasonal malaria chemoprevention (SMC), data collection was split into two stages: July 2017, prior to the SMC campaign (during rainy season and the start of the high transmission season), and from September to November 2017, concurrent with the SMC campaign (after the rainy season, the highest transmission period). The sample size was calculated according to the objectives of the recall validation study [27]. In total 1600 U5s with current or recent fever were required: 200 who received ACT, and 200 who did not receive ACT, at each of the four types of recruitment site (public urban, public rural, private urban and CHW).

### Data analysis

Data were collected on paper questionnaires then double-entered into a CSPro template, with discrepancies resolved by reviewing the original questionnaires. Data cleaning and analysis was completed in Stata version 14 and R version 3.5. Drug names and brands recorded by health workers that were not pre-coded in the consultation questionnaire were classified by first searching the ACTwatch database of antimalarial drugs [29], then using broader internet searches if not found. Drugs not prescribed for malaria were classified according to general purpose (e.g. antihistamine, antibiotic). The drug formulation type (e.g. tablet, syrup, injectable) was not systematically recorded in the questionnaire, but was classified as oral (tablet, syrup, suspension), injectable (intravenous or intramuscular) or unknown formulation (not recorded on consultation questionnaire) during data processing based on available information.

Descriptive analysis to assess differences in case management practices between facility types used Chi-squared tests, or Fishers’ exact test if any cell value was less than ten. To describe the classification of prescribed treatment as in agreement or disagreement with Mali malaria case management practices, Sankey diagrams were prepared using the networkD3 package in R. Denominator values are presented in all results tablets to indicate any instances where specific indicators were missing from data collection forms.

Correctly managed cases were defined as children who had a negative test result and did not receive any antimalarial, or children with a positive test who received ACT and no other antimalarial. Children who were not tested by RDT or microscopy, who received any antimalarial drugs (ACT or non-ACT) after a negative test result, or who had a positive test result but received drugs other than as recommended in the case management guidelines (a combination of ACT and non-ACT antimalarials, or non-ACT antimalarials only) were classified as incorrectly managed.

To explore factors associated with incorrectly managed cases, mixed-effect logistic regression models were developed using the binary classification of incorrect or correct case management as the primary outcome and including a random intercept for each recruiting site. A backward stepwise approach was taken, whereby all potential covariates were initially included, then the least significant variable dropped until all remaining variables were *p* < 0.100. Use of *p* < 0.100 to assess covariates for logistic regression modelling is standard practice, since more restrictive *p* values 0.05 have been shown to fail to identify known relevant variables [30]. Caregiver age, sex, and level of education, age, sex, and axillary temperature of child, type of health facility visited, if consultation was on a weekday (Monday – Friday) or weekend (Saturday – Sunday), and season (early transmission season or peak transmission season) were tested in models. To allow investigation of factors specifically associated with drug prescriptions, additional models were developed for the subset of children who had received confirmatory testing. Models were also developed for the outcome of receipt of severe malaria drugs: injectable artemether, artesunate or quinine, irrespective of health worker diagnosis or confirmatory testing. Since this study was completed as a secondary analysis of existing data, covariates available for testing in models were limited to those available in the existing dataset. Selection of covariates was informed by review of existing literature on malaria case management and health worker performance.

## Results

### Study participants

A total of 1602 children under 5 years were recruited while attending participating health facilities seeking diagnosis and treatment for febrile illness (Table 1). Overall, 54.4% of children were male, and similar proportions of male and female children were recruited by age. Nearly all caregivers taking children to CHWs were female (94.4%), while public rural facilities had the largest proportion of male accompanying caregivers of all site types (30.5%). Nearly half of all caregivers were aged 25–34 (46.3%). Caregivers’ level of education varied according to recruiting health facility type and urban or rural location: while the majority of caregivers in rural areas reported no formal education (80.4% attending CHWs, 66.8% attending public rural facilities), it was common for urban caregivers to have secondary or higher education levels (47.7% attending public urban, 72.3% attending private).

**Table 1:** Demographics of enrolled febrile children and their caregivers, according to site of enrolment (public health facility in urban area, public health facility in rural area, community health worker (CHW) or private facility in urban area)

	All sites		Public Urban		Public Rural		CHW		Private Urban	
	n	%	n	%	n	%	n	%	n	%
Sex of sick child (*N* = 1602)										
Male	872	54.4%	228	54.2%	236	56.2%	222	52.2%	186	55.4%
Female	730	45.6%	193	45.8%	184	43.8%	203	47.8%	150	44.6%
Age of sick child in years (*N* = 1602)										
< 1	305	19.0%	69	16.4%	76	18.1%	100	23.5%	60	17.9%
1	329	20.5%	107	25.4%	75	17.9%	55	12.9%	92	27.4%
2	367	22.9%	103	24.5%	84	20.0%	99	23.3%	81	24.1%
3	287	17.9%	70	16.6%	94	22.4%	61	14.4%	62	18.5%
4	314	19.6%	72	17.1%	91	21.7%	110	25.9%	41	12.2%
Sex of caregiver (*N* = 1602)										
Male	289	18.0%	82	19.5%	128	30.5%	24	5.6%	55	16.4%
Female	1313	82.0%	339	80.5%	292	69.5%	401	94.4%	281	83.6%
Caregiver age in years (*N* = 1602)										
18–24	416	26.0%	112	26.6%	118	28.1%	113	26.6%	73	21.7%
25–34	742	46.3%	200	47.5%	174	41.4%	203	47.8%	165	49.1%
35–44	330	20.6%	83	19.7%	83	19.8%	78	18.4%	86	25.6%
≥ 45	114	7.1%	26	6.2%	45	10.7%	31	7.3%	12	3.6%
Caregiver education level (*N* = 1600)										
None	751	46.9%	102	24.2%	280	66.8%	341	80.4%	28	8.3%
Primary	382	23.9%	118	28.0%	122	29.1%	77	18.2%	65	19.3%
Secondary or higher	467	29.2%	201	47.7%	17	4.1%	6	1.4%	243	72.3%

### Reported use of parasitological tests and adherence to test results

According to records of consultation made by the attending health workers, a confirmatory malaria test was not performed consistently among the recruited children (Table 2). While 83.6% of children recruited at public urban facilities and 66.6% recruited at CHW site were tested for malaria, use of parasitological testing was infrequent at public rural (27.0%) and private urban sites (13.7%). Among all children who received a malaria test and the result was recorded, 72.1% had a positive result. There is limited evidence for higher test positivity in the peak transmission season (76.0% in peak season versus 70.1% in early season, *p* = 0.066). There were some differences in use of confirmatory tests by season in some facility types, with the proportion of recruited children receiving an RDT or microscopy decreasing in the peak season compared to early transmission season at public rural facilities (34.3% in early to 19.0% in peak, *p* < 0.001) and CHW sites (83.5% in early to 50.3% in peak, *p* < 0.001). Comparing weekly testing rates at the study facilities with data from the Mali Health Commodity Dashboard (OSPSANTE) indicated that while there was some evidence of low stock or stockout of RDTs, there remained facilities and time periods where testing was low but no RDT stock issues were reported (Supplementary File 2).

**Table 2:** Diagnostic procedures performed on enrolled febrile children by health facility type, and by transmission season (early transmission season: July, or peak season: September to November)

	Type of recruiting health facility								Transmission season			
	Public Urban		Public Rural		CHW		Private Urban		Early season		Peak season	
	n	%	n	%	n	%	n	%	n	%	n	%
Diagnostic method (*N* = 1601)												
No diagnostic method used	0	0.0%	0	0.0%	0	0.0%	24	7.1%	6	1.0%	18	1.8%
Clinical signs/symptoms only	69	16.4%	306	73.0%	142	33.4%	266	79.2%	253	41.6%	530	53.4%
Rapid diagnostic test	101	24.0%	112	26.7%	283	66.6%	11	3.3%	214	35.2%	293	29.5%
Microscopy	251	59.6%	1	0.2%	0	0.0%	35	10.4%	135	22.2%	152	15.3%
Axillary temperature (*N* = 1602)												
Not measured	37	8.8%	18	4.3%	20	4.7%	17	5.1%	32	5.3%	60	6.0%
< 37.5 °C	143	34.0%	60	14.3%	136	32.0%	146	43.5%	189	31.0%	296	29.8%
37.5–38.4 °C	99	23.5%	193	46.0%	148	34.8%	101	30.1%	213	35.0%	328	33.0%
38.5–39.4 °C	101	24.0%	97	23.1%	102	24.0%	58	17.3%	130	21.4%	228	23.0%
≥ 39.5 °C	41	9.7%	52	12.4%	19	4.5%	14	4.2%	45	7.4%	81	8.2%
Test result, among those tested (*N* = 794)												
Negative for malaria	88	25.0%	14	12.4%	101	35.7%	12	26.1%	83	23.8%	132	29.7%
Positive for malaria	264	75.0%	99	87.6%	179	63.3%	31	67.4%	263	75.4%	310	69.7%
Invalid	0	0.0%	0	0.0%	3	1.1%	0	0.0%	2	0.6%	1	0.2%
Don’t know result	0	0.0%	0	0.0%	0	0.0%	3	6.5%	1	0.3%	2	0.5%

Health workers were asked to report their final diagnosis for each child (malaria or not malaria). While health workers agreed with all positive malaria test results, a final diagnosis of malaria was made for nearly a quarter (21.4%) of all children with a negative test result. Discrepancies between parasitological test result and health worker diagnosis occurred primarily at public urban facilities (40.9% of 88 test-negative children diagnosed as malaria by the health worker) (Table 3). The proportion of all test-negative children who were diagnosed as malaria by the health worker increased in the peak transmission season (from 14.5 to 26.0% of all children with negative test, *p* = 0.046). However, the proportion of children with negative test results which were diagnosed as malaria by the health worker did not differ according to whether the child was tested by RDT or microscopy (*p* = 0.39).

**Table 3:** Agreement between diagnostic test result and health worker’s stated final diagnosis

	Type of recruiting health facility								Transmission season			
	Public Urban		Public Rural		CHW		Private Urban		Early season (July)		Peak season (Sept - Nov)	
	n	%	n	%	n	%	n	%	n	%	n	%
Among those with negative malaria test result (*N* = 214)												
Diagnosed as malaria by health worker	36	40.9%	4	28.6%	4	4.0%	2	16.7%	12	14.5%	34	26.0%
Diagnosed as not malaria by health worker	52	59.1%	10	71.4%	96	96.0%	10	83.3%	71	85.6%	97	74.1%
Among those with positive malaria test result (*N* = 573)												
Diagnosed as malaria by health worker	264	100%	99	100%	179	100%	31	100%	263	100%	310	100%
Diagnosed as not malaria by health worker	0	0%	0	0%	0	0%	0	0%	0	0%	0	0%
Among those not tested by RDT or microscopy (*N* = 795)												
Diagnosed as malaria by health worker	47	68.1%	291	96.0%	94	70.7%	153	52.8%	192	76.5%	393	72.2%
Diagnosed as not malaria by health worker	22	31.9%	12	4.0%	39	29.3%	137	47.2%	59	23.5%	151	27.8%

Among children who were not tested, almost all (96.0%) were presumptively diagnosed as malaria at public rural facilities, while at public urban facilities just over half (52.8%) of febrile children who did not receive a test were presumed to have malaria (Table 3). The proportion of untested children diagnosed as malaria cases by health workers did not differ by transmission season (*p* = 0.26).

#### Treatment prescribed

**Fig. 1:**
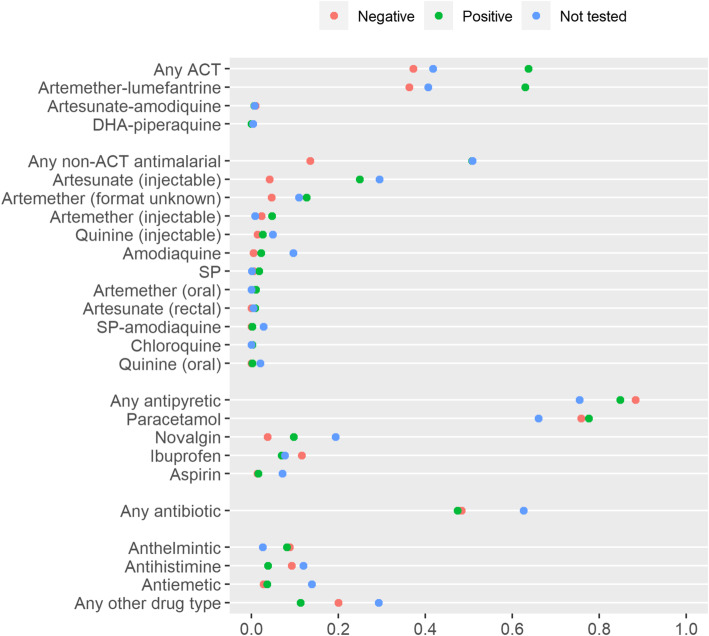
Proportion of children receiving specific drugs across all sites, summarized by parasitological test result. Parasitological test result determined by microscopy or rapid diagnostic test. Equivalent figures describing receipt of drugs by test result within each specific health facility type can be found in Supplementary File 4

Of the 573 children with test-confirmed malaria, nearly two-thirds (63.7%) received an ACT (Fig. 1). The most commonly prescribed ACT was the first-line treatment AL (98%), with a small proportion of children receiving second-line AA or dihydroartemisinin-piperaquine. 106 (18.5%) children with confirmed malaria received non-ACT antimalarials in addition to an ACT (Supplementary File 3). Overall, more than half of children with confirmed malaria (50.8%) received an antimalarial that was not an ACT. Prescription of antimalarials other than ACTs was reported at all types of health facility, even though children with signs of severe malaria were excluded from the study. The most commonly reported non-ACT antimalarials prescribed were artesunate injection and artemether of unspecified formulation (but likely to be injection).

Antibiotics were prescribed to more than half (58.5%) of children with confirmed malaria (48% oral, 21% injectable, 20% unknown formulation, 11% multiple formulations). Antipyretic drugs were prescribed to most (84.8%) of children with confirmed malaria; most commonly paracetamol (92%), with small numbers receiving ibuprofen (8%), aspirin (2%), or metamizole (Novalgin, 12%).

Among 215 children with a negative malaria test result, 44.2% received any type of antimalarial: 69.5% ACT, 15.8% non-ACT, and 14.7% both ACT and non-ACT. Similar to the children with confirmed malaria, the most commonly reported non-ACTs prescribed to those with a negative test were artesunate injection and artemether (assumed injection) (Fig. 1). Antibiotics were prescribed to 60.9% of children with negative malaria test result (65% oral, 12% injectable, 17% unknown formulation, 6% multiple formulations). Prescription of antipyretics to children with negative malaria test was high: 88.4% received any antipyretic, with paracetamol the most commonly prescribed. Figures describing drugs received by testing status at each recruiting health facility type are provided in Supplementary File 4.

Of the 797 children who did not receive a parasitological test for malaria, 75.4% received at least one type of antimalarial: 32.6% ACT, 44.6% non-ACT, and 22.8% received both ACT and non-ACT. Artesunate injection and artemether (assumed injection) were the non-ACTs most frequently prescribed to children who were not tested, however 9.7% of children without a malaria test received amodiaquine (Fig. 1). More than two-thirds (69.0%) of children who were not tested received an antibiotic.

It was common for children in the study to receive multiple drugs during their consultation: 21.7% of all children in the study received more than one type of antimalarial, 14.0% received more than one type of antibiotic, and 51.9% received both an antibiotic and an antimalarial. Prescription of multiple antimalarials or multiple antibiotics was much more common at public rural sites, accounting for 71 and 84% of instances observed, respectively. Prescribing both an antibiotic and an antimalarial occurred less frequently at CHW and private sites (24 and 29% of children, respectively) than at public urban and public rural facilities, where 67 and 83% of all febrile U5s received both an antibiotic and an antimalarial. Use of injectable antimalarials (quinine, artemether or artesunate; excluding artemether or artesunate of unknown formulation) was most common at public rural facilities, where 52% of all participating children received an injectable antimalarial, compared to 35% at public urban sites, 16% at CHW sites, and 15% at private facilities.

**Fig. 2:**
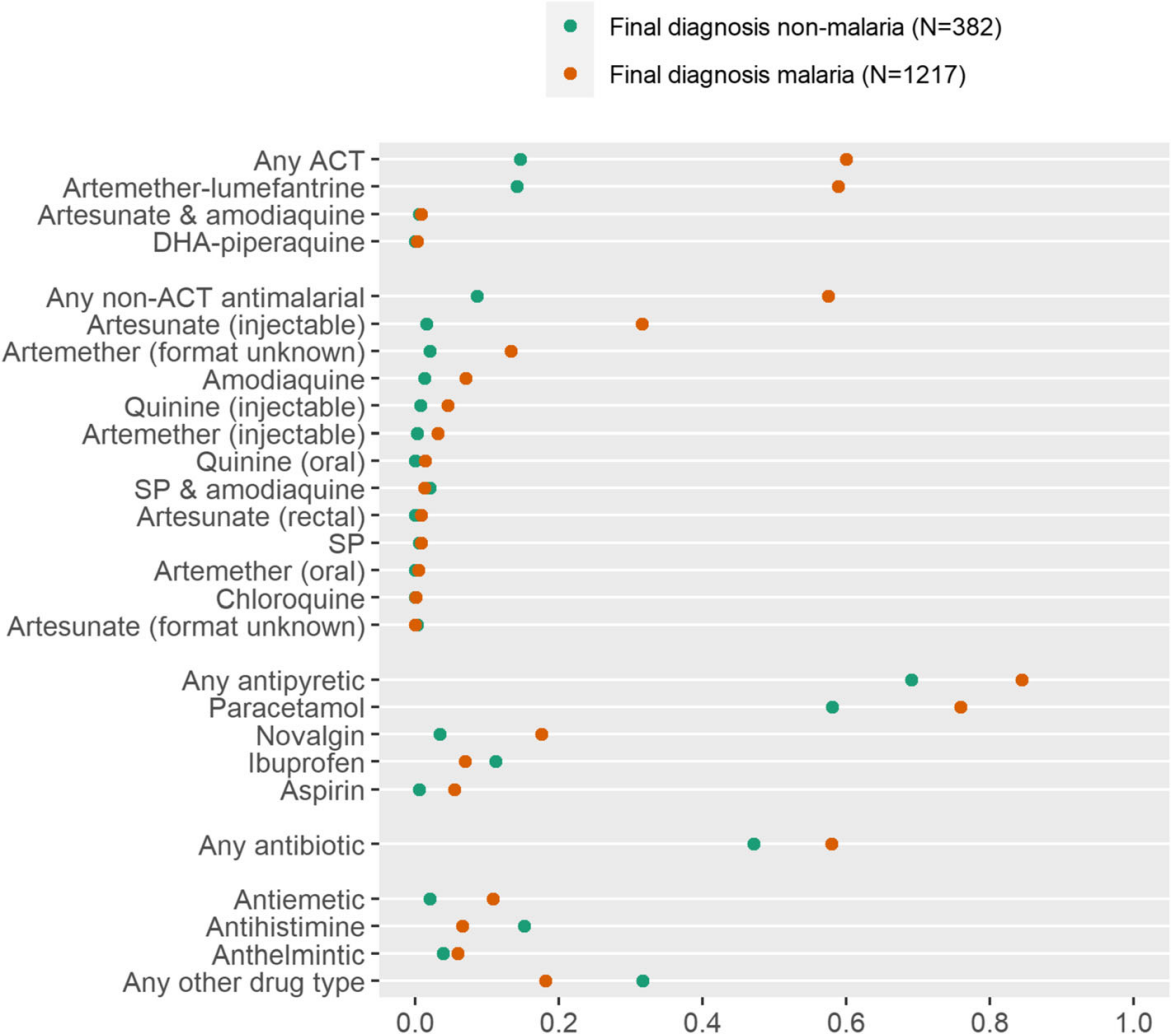
Proportion of all children receiving specific drugs across all sites, summarized by health worker diagnosis. Data are summarized by final diagnosis made by the health worker (malaria or non-malaria), irrespective of parasitological test result

Drugs prescribed according to the diagnostic decision made by the health worker were not necessarily in agreement with any parasitological test results (Fig. 2). Health workers did not provide ACT to all children that they believed to have malaria, and antimalarials continued to be prescribed to children even when then health worker’s diagnosis was that the child did not have malaria.

#### Adherence to malaria case management policies

**Fig. 3:**
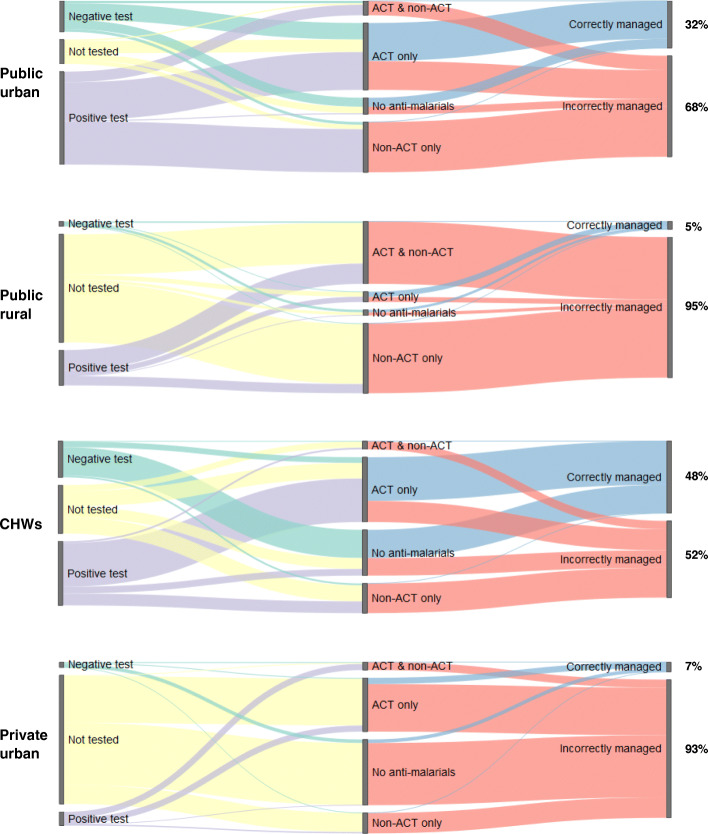
Sankey flowchart summarizing key case management steps at each type of health facility. The height of vertical grey bars indicates the proportion of children at each facility type who were tested, had negative or positive test result (left bars), the proportion of children receiving specific types of drugs (central bars), and the proportion of children correctly or incorrectly managed (right bars). Colored bars indicate the proportion of children moving from one category to the next, e.g. the proportion who had a negative test and received ACT. The final classification of correct or incorrect management is conditional on both diagnostic testing and type of drug(s) prescribed

Across all sites, less than a quarter (23.7%) of children recruited were classified as appropriately managed during their consultation, defined according to receipt of diagnostic test and the drugs prescribed following the test result. Flow diagrams (Fig. 3) illustrate the proportion of children who were correctly and incorrectly managed at each type of site; linking their RDT or microscopy test result with the types of drugs they received and the classification of correct or incorrect case management. The largest proportion of children appropriately managed were those recruited at CHW sites (48.4%), while 31.8% were managed correctly at public urban sites. Only 5.3% of children recruited from public rural sites and 6.6% recruited from private facilities were defined as correctly managed: incorrect management was primarily a result of failure to perform a diagnostic test. At public urban facilities, the most common reason for incorrect management was prescription of a non-ACT antimalarial only following a positive test result (29.2% of all children recruited). Incorrect case management at CHW sites resulted from a range of different situations including lack of testing, prescribing no antimalarials after a positive test result, or prescribing ACT after a negative test result.

Exploring data from the routine commodity management system (OSPSANTE) indicated that there were some stockouts of one or both doses of AL suitable for U5s at two public facilities, which could have contributed to use of non-ACTs at these facilities, however, the other two public facilities had no evidence of AL stockouts but prescribed non-ACTs throughout the study period, suggesting that use of non-ACTs in the study population cannot be solely attributed to drug stockouts (Supplementary File 5). A full description of correct and incorrect management at different stages of the consultation for each type of recruiting health facility is provided in Supplementary File 6.

#### Factors associated with correct management

Where correct management of uncomplicated malaria was defined as either receiving ACT (without non-ACT antimalarials) following a positive confirmatory test or receiving no antimalarials after a negative confirmatory test, and incorrectly managed cases included those who were not tested, received ACT after a negative test or received a non-ACT antimalarial after either positive or negative test result, incorrect case management was more common in the peak transmission season, in public urban facilities, and on weekends (Table 4). Some associations were also seen with caregiver age, where children of caregivers aged 35–44 years were more likely to have received incorrect management than children of caregivers aged 18–24 years (Table 4).

**Table 4:** Multivariate models describing factors associated with incorrect case management

	Odds of incorrect case management of febrile children under 5 years					
	Model 1: Children not receiving diagnostic test considered as ‘incorrectly managed’ (*N* = 1510)			Model 2: Children not receiving diagnostic test excluded from model (*N* = 742)		
Variable	OR	95% CI	p	OR	95% CI	p
Type of health facility						
Public urban	1.00	–	–	1.00	–	–
Public rural	20.75	0.96, 447.4	0.053	2.76	0.62, 12.24	0.182
Community health worker	0.49	0.05, 4.72	0.536	0.15	0.5, 0.45	0.001
Private	2.16	0.18, 25.89	0.542	0.30	0.08, 1.16	0.080
Day of visit to health facility						
Weekday (Monday – Friday)	1.00	–	–	1.00	–	–
Weekend (Saturday – Sunday)	1.71	1.09, 2.68	0.019	1.73	1.03, 2.88	0.037
Transmission season						
Early transmission season (July)	1.00	–	–			
Peak transmission season (September–November)	1.32	0.96, 1.81	0.091			
Caregiver age						
18–24	1.00	–	–			
25–34	1.23	0.88, 1.74	0.228			
35–44	1.59	1.03, 2.44	0.035			
≥45	1.42	0.75, 2.69	0.286			
Caregiver sex						
Female				1.00	–	–
Male				1.52	0.93, 2.48	0.092
Axillary temperature of child (increasing)	1.38	1.18, 1.61	< 0.001	1.77	1.48, 2.13	< 0.001

Correct management was defined in both models as receipt of ACT but no non-ACT antimalarials following a positive test, or receipt of neither ACTs nor non-ACT antimalarials following a negative test. Incorrect management in both models included receipt of non-ACT antimalarial following either as positive or negative test, or receipt of ACT following a negative test. In model 1, children who did not receive a parasitological test were considered as incorrectly managed; in model 2 children who did not receive any parasitological test were excluded

If children who did not receive RDT or microscopy testing were excluded from the definition of correct/incorrect management, factors associated with incorrect management among remaining children (*N* = 742) included being taken to the facility by a male caregiver, attending a public rural health facility, and attending on a weekend (Table 4). Incorrect case management by both definitions became more common with increasing measured axillary temperature.

Receipt of injectable antimalarials was found to be associated with axillary temperature of the child, where children with higher temperature had increased odds of receipt of injectable antimalarials, and also to be more common among children who either were not tested by RDT or microscopy or had a positive test compared to those with a negative test (Table 5). An association was also observed between increasing child age and increasing odds of injectable antimalarial prescription.

**Table 5:** Multivariate model (*N* = 1510) describing factors associated with receipt of severe malaria drugs

Variable	OR	95% CI	p
Type of health facility			
Public urban	1.00	–	–
Public rural	6.34	0.15, 266	0.333
Community health worker	0.07	0.003, 1.35	0.078
Private	0.16	0.01, 4.20	0.273
Parasitological testing			
Negative test result	1.00	–	–
Positive test result	4.84	2.84, 8.26	< 0.001
Not tested	3.65	2.03, 6.56	< 0.001
Axillary temperature of child			
< 37.5 °C	1.00	–	–
37.5–38.4 °C	2.30	1.61, 3.28	< 0.001
38.5–39.4 °C	4.40	2.94, 6.59	< 0.001
≥ 39.5 °C	8.78	4.85, 15.89	< 0.001
Age of child in years (increasing)	1.24	1.11, 1.38	< 0.001

Severe malaria drugs included are injectable artemether, artesunate or quinine. Where reported drug was artemether with formulation unspecified (*n* = 171), the formulation was assumed to be injectable

## Discussion

This study provides a snapshot of management of malaria in febrile children U5 in Mali at urban private and public health facilities and at rural public facilities and community health worker sites. While the primary aim of the original data collection was to explore validity of caregiver recall of drugs received during consultations [27], this secondary analysis explored malaria diagnostic procedures and medications prescribed in further detail, aiming to describe adherence to national malaria diagnosis and treatment guidelines and to explore factors associated with deviation from guidelines. Use of parasitological testing among the study population of febrile children U5 was inconsistent, with particularly low testing rates at public facilities in rural areas and private facilities in urban areas. The most common deviation from national treatment guidelines was prescription of antimalarial drugs other than ACTs; drugs such as artesunate injection or artemether were given to some children in combination with ACT, and to others in place of ACT. When considering both diagnostic and treatment procedures, more than three-quarters of enrolled febrile children under 5 years received inappropriate malaria case management during their consultation.

Use of parasitological testing varied by type of site, with low testing rates at public rural and private urban facilities in the study. There was some evidence of a decrease in confirmatory testing in the peak transmission season (September–October) compared to the early transmission season (July), which could be attributable to health worker assumptions that fever is more likely attributable to malaria in the peak season, or simply a result of increased patient load and limited human resources for testing. OPSANTE data indicate that while some study facilities experienced low stock or stockouts of RDTs during the study period, this does not fully explain low use of parasitological testing at other facilities and time periods with no apparent RDT stock problems. Failure to use parasitological diagnosis has been identified as one of the main gaps in malaria case management in several other settings [13–15, 31, 32], with some hypothesizing that high patient load at facilities has a detrimental impact on diagnostic testing rates [13, 15].

In addition to recording microscopy or RDT result, the study collected the health worker’s final diagnosis of malaria or not malaria. Discrepancies where a test was negative but the health worker diagnosed malaria were observed primarily at the public urban health facilities. While there was no association between this type of discrepancy and type of diagnostic, we cannot exclude that there was a lack of trust in both RDT and microscopy, or that specific clinical presentations led to the health worker disagreeing with the test result. While data were not collected on characteristics of health workers, odds of incorrect management were lower at CHW sites than public urban and rural sites, and evidence from other settings indicates that more senior health workers are more likely to deviate from standard case management guidelines [17]. Interestingly, when reviewing prescription according to the health worker’s diagnosis of malaria or not malaria, irrespective of diagnostic test result, health workers did not consistently give ACT to all children they ultimately diagnosed as malaria cases, and some children that they thought did not have malaria did received antimalarials. This indicates that lack of adherence to treatment guidelines is not solely due to lack of trust in the diagnostic test result, but may reflect health worker or caregiver preference for antimalarial drugs, or a desire to provide antimalarial treatment even if malaria is not thought to be primary cause of current febrile illness. Other studies have found that individual health worker training is more influential in determining adherence to guidelines than commodity supply issues [33], but the need for health worker autonomy to deviate from guidelines was highlighted in a study in Madagascar [34], where clinicians justified prescribing antimalarial to test-negative individuals who may have self-treated prior to attending the facility. Patient flow has also been shown to be relevant in some settings, where drugs may be prescribed before laboratory test results are available [35].

Non-ACT antimalarials were prescribed to febrile children U5 participating in this study, most commonly drugs recommended for treatment of severe malaria: injectable artesunate, artemether or quinine. Children with signs of severe malaria or other severe illness were not eligible for inclusion in the study, and while there is a possibility that children with signs of severe malaria were erroneously included, it is very unlikely that this can explain the full extent of injectable antimalarials in this setting. It is not possible to determine the precise motivations for prescription of non-ACT antimalarials in the current study, but injectables were more commonly prescribed to older children (within the study range of one to 59 months) and those with higher axillary temperatures. It is possible that health workers and caregivers are concerned about severe malaria in this high transmission setting and consequently take a cautious approach to treatment of malaria in under-fives, choosing to give drugs for severe malaria to those perceived to be at higher risk of progression to severe disease. The use of injectables could also be explained by caregiver or clinician preferences for injectables over oral drugs [34]. The use of injectable antimalarials in the current study is in contrast to findings from other settings. ACT Consortium studies across five countries found a substantial proportion of patients with confirmed uncomplicated malaria receiving drugs other than ACTs; but most commonly amodiaquine, chloroquine or SP rather than injectable antimalarials [19]. Two studies in Sudan identified relatively high proportions of children with uncomplicated malaria receiving injectable quinine [36], but with receipt of injectables more frequent in those over five than under 5 years [37]. Monotherapy was also more common in those aged older than five in studies in Nigeria [15] and Equatorial Guinea [18].

When monotherapy is prescribed for severe malaria, parenteral drugs should be given for a minimum of 24 h and always followed by a full oral course of ACT [4, 38]. While some children in this study were prescribed both ACT and an injectable, some received only the injectable. No follow up data are available to determine if children given injectable monotherapy subsequently returned to the facility for a course of ACT. A further factor that should be considered when interpreting prescription practices is the cost-recovery model used at health facilities in Mali. Both diagnostic testing and ACT are free for children under 5 years, but other antimalarial drugs and injectable drugs generally require payment. Drug and service charges support the facility operating costs, therefore there may be economic motivations from health workers to prescribe additional drugs for children under five with malaria. Prescribing artemisinin monotherapies intended for severe malaria drugs to patients with uncomplicated malaria is a concern due to risk of drug resistance [39, 40], particularly when injectable monotherapy is not followed by a full course of ACT, but also has immediate consequences in increased wastage and drug costs for the facilities, and represents an increased risk to the patient due to use of an invasive treatment when safe and effective oral drugs are available.

Polypharmacy was common in this study, particularly the prescription of an antimalarial drug together with an antibiotic. This finding likely reflects the challenge of managing febrile illness in children in a high malaria transmission setting, where *P. falciparum* parasite rate by RDT in children aged six to 59 months was estimated at 25.5% in 2015 [10]. In such settings, health workers must assess if clinical signs and symptoms are due to malaria or another cause. Prescription of multiple drugs for uncomplicated malaria was found to be common in Nigeria, with antimalarials often prescribed in combination with analgesics, antibiotics or vitamin preparations, leading to concerns about potential drug interactions [15]. In Tanzania, U5s were more commonly prescribed antimalarials and antibiotics together than older children [41], while a study in Burkina Faso found that antibiotics are prescribed to 84% of all U5s attending rural public health facilities in the dry season [42]. Challenges remain in management of febrile illness in the tropics, particularly in rural areas with limited diagnostic capacity [43]. In the current study, the prescription of antimalarials to children with negative malaria test could be an indication of lack of alternative diagnostic tools to determine fever etiology, consequently these children may not be treated for the true cause of their fever. Antibiotic prescription has been shown to increase following scale up of RDTs and increased malaria testing, however, this has prompted concerns regarding use of appropriate antibiotics for the most common bacterial causes of fever, and over-use of antibiotics promoting development of antimicrobial resistance [44]. Identification of biological markers of severe disease or likelihood of progression to severe disease could be of great value, allowing health workers to identify children who require rapid referral, admission or supportive care [45]. Ongoing research using electronic decision trees in combination with point of care tests and clinical signs show promise in improving health workers’ ability to identify severe disease and prevent unnecessary antibiotic prescription [46], but further evidence is required.

Description of malaria case management practices in Mali was not the primary aim of the study which collected these data, therefore the findings presented are limited by failure to collect additional indicators which would have been of interest to the current analysis. Description of health worker characteristics (gender, qualifications, years of experience, role at facility), consistent recording of drug formulation type or administration route, and stock of malaria drugs and diagnostic materials at the facilities could have added useful detail to the current study and aided interpretation of results. Additional description of patient clinical signs and comorbidities would also have aided interpretation of drug choices made by health workers, particularly in relation to co-prescription of antimalarials and antibiotics, or the use of injectable antimalarials. All data describing diagnostic method used, test results and drugs prescribed were recorded by the health worker without independent validation. Additionally, no follow up data were collected to determine if children given injectable monotherapy subsequently returned to the facility for a full course of ACT. While study team members were present at the facilities, they did not observe the consultation or conduct exit interviews with caregivers to avoid introducing bias in the primary study assessing caregiver recall of treatment, and there was no independent validation that children with signs of severe disease were excluded. Finally, this study is limited by the relatively small number of health facilities and health workers participating, therefore the findings may not be generalizable to the broader context in Mali.

## Conclusions

Multiple limitations in management of febrile children under five were identified: in use of diagnostics, adherence to test results, and in selection of drugs to prescribe. Under-use of diagnostic testing appeared to be particularly common in specific facilities and may be linked with workload or supply chain difficulties. Other leading concerns for further investigation include the prescription of antimalarial drugs to patients tested negative by RDT or microscopy, and the frequent prescription of non-ACTs such as injectable artesunate and artemether either in place of or in combination with ACT. It is unclear if the high use of non-ACTs is influenced by provider or caregiver preferences and perceived effectiveness, or by concerns about potential for children under five to develop severe malaria. Severe malaria drugs are more expensive than ACTs and are procured in smaller quantities. Their over-use is costly for the program, but importantly also risks stock-outs for future children seeking care with severe malaria. Increased use of monotherapies, particularly when not followed by a full course of ACT, risks promoting development of drug resistance. Future qualitative research could help to understand the drivers of antimalarial drug selection by clinicians and drug preferences of both clinicians and caregivers, to guide strategies for improvement of case management in this setting.

## Supplementary Information

**Additional file 1.** Questionnaire used to record consultation details at health facility or community health worker site.

**Additional file 2.** Summary of testing by RDT and microscopy at public facility sites by study week, and corresponding OPSANTE data describing periods of RDT stockout.

**Additional file 3.** Description of case management practices at all sites and correct/incorrect case management designation.

**Additional file 4.** Proportion of children receiving specific drugs, described by each recruiting facility type.

**Additional file 5.** Summary of antimalarial drug prescription at public facility sites by study week, and corresponding OPSANTE data describing periods of ACT stockout.

**Additional file 6.** Description of case management practices by study site type and correct/incorrect case management designation.

## Data Availability

The data analyzed in the current study are available from the corresponding author on reasonable request.

## Abbreviations

AA: Artesunate-amodiaquine
ACT: Artemisinin combination therapy
AL: Artemether-lumefantrine
AQ: Amodiaquine
CHW: Community health worker
CSCom: Community health center (*Centres de Santé Communautaire*)
CSRef: Referral health center (*Centres de Santé de Réference*)
EUV: End user verification survey
RDT: Rapid diagnostic test
SMC: Seasonal malaria chemoprevention
SP: Sulfadoxine-pyrimethamine
U5: Children under five years
