# Supplementary material for: Management of uncomplicated malaria among children under five years at public and private sector facilities in Mali
Source: BMC Public Health. 2020 Dec 9;20:1888. doi: 10.1186/s12889-020-09873-1 (PMC7724888; doi:10.1186/s12889-020-09873-1)
Supplement: Supplementary file 1 — Additional file 1. Questionnaire used to record consultation details at health facility or community health worker site. [file 12889_2020_9873_MOESM1_ESM.pdf]

TULANE UNIVERSITY / INFOSTAT VERSION DATE: 04 JULY 2017

CONSULTATION RECORD AT HEALTH FACILITY/COMMUNITY HEALTH WORKER

A

IDENTIFICATION

FACILITY NAME ______________________________

DATE OF CONSULTATION . . . . [D][D] [M][M] [Y][Y]

FACILITY CODE . . . . [ ][ ]

CAREGIVER CODE . . . . [ ][ ][ ]

UNIQUE CAREGIVER ID . . . . [ ][ ][ ][ ][ ]

SECTION 1. CONSULTATION RECORD

NO.	QUESTIONS AND FILTERS	CODING CATEGORIES	SKIP
101	Name child	______________________________	
102	Is [child] male or female?	MALE . . . . 1<br>FEMALE 2	
103	How old is [child]?<br><br>(IF LESS THAN ONE YEAR OLD, ENTER 00 YEARS AND COMPLETE AGE IN MONTHS)	AGE IN COMPLETED YEARS . . . . [ ][ ]<br><br>If <1 year:<br>AGE IN COMPLETED MONTHS . . [ ][ ]	
104	What is the axillary temperature (in °C)?	[ ][ ] . [ ]<br>If not measured, enter 99.9	
105	What diagnostic method was used?	NONE . . . . 0<br>CLINICAL ONLY . . . . 1<br>RAPID DIAGNOSTIC TEST . . . . 2<br>MICROSCOPY . . . . 3<br><br>OTHER ______________ 8<br>*(specify)*	
106	What is the diagnosis made by the health provider?	MALARIA . . . . 1<br>NOT MALARIA 0	
107	What was the result of the malaria diagnostic	NEGATIVE . . . . 0<br>POSITIVE . . . . 1<br><br>NOT TESTED . . . . 7<br>INVALID . . . . 8<br>DON'T KNOW 9	

NO.	QUESTIONS AND FILTERS	CODING CATEGORIES	SKIP
108	What drugs were prescribed or given to the sick child?<br><br>(LIST ALL DRUGS PRESCRIBED/GIVEN)	**ANTIMALARIALS**<br>ARTEMETHER-LUMEFANTRINE (COARTEM) A<br>ARTESUNATE-AMODIAQUINE . . . . B<br>SP . . . . C<br>SP-AMODIAQUINE . . . . D<br>CHLOROQUINE . . . . E<br>AMODIAQUINE . . . . F<br>QUININE PILLS . . . . G<br>QUININE INJECTION IV/IM . . . . H<br>ARTESUNATE RECTAL . . . . I<br>ARTESUNATE INJECTION IV/IM . . . . J<br>OTHER ANTIMALARIAL . . . .<br>________________ K<br>*(specify)*<br>**ANTIBIOTICS**<br>AMOXICILLIN . . . . L<br>ANTIBIOTIC PILLS / SYRUP . . . . M<br>ANTIBIOTIC INJECTION . . . . N<br>ASPIRIN . . . . O<br>PARACETAMOL / ACETAMINOPHEN . . . . P<br>IBUPROFEN . . . . Q<br>KUNBILENI . . . . R<br>KUNBILENI JEMAN . . . . S<br><br>OTHER NOT LISTED: ________________ X<br>*(specify)*<br>DON'T KNOW . . . . Z	
109	Where do you intend to collect these drugs?	DRUGS COLLECTED AT FACILITY . . . . 1<br>DRUGS COLLECTED FROM ELSEWHERE . . 2<br>DEPENDS ON PATIENT PREFERENCE . . . . . 3<br>DON'T KNOW 9	
