# Supplementary material for: Management of uncomplicated malaria among children under five years at public and private sector facilities in Mali
Source: BMC Public Health. 2020 Dec 9;20:1888. doi: 10.1186/s12889-020-09873-1 (PMC7724888; doi:10.1186/s12889-020-09873-1)
Supplement: Supplementary file 2 — Additional file 2. Summary of testing by RDT and microscopy at public facility sites by study week, and corresponding OPSANTE data describing periods of RDT stockout. [file 12889_2020_9873_MOESM2_ESM.pdf]

Supplementary File 2

Fomba et al. Management of uncomplicated malaria among children under five years at public and private sector facilities in Mali

Weekly proportion of participants tested by microscopy, rapid diagnostic test (RDT), or not tested at public urban and public rural sites, with indication of when RDT stockouts likely occurred, according to OSPSANTE data

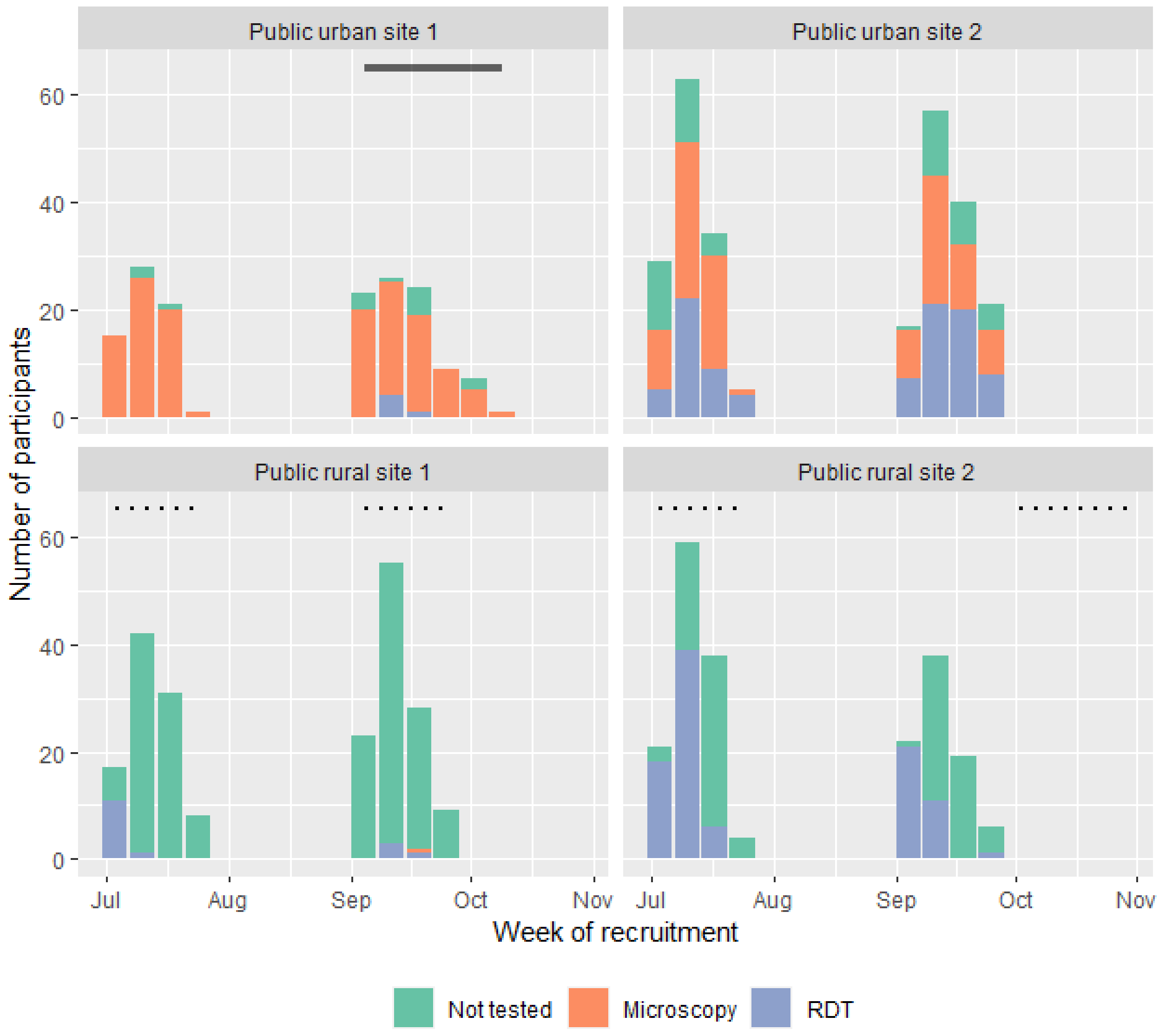

<u>Data used</u>

The above figure combines data collected as part of the study with routinely collected data in the Health Commodity Dashboard system used in Mali (OSPSANTE). Study data collected from the two public urban facilities and two public rural facilities participating in the study describe the proportion of recruited febrile children under five who were tested using microscopy, using RDT, or not tested, during each week of the study.

Solid black horizontal lines indicate a month in which the OSPSANTE system recorded that the facility had zero RDTs in stock. Dotted black horizontal lines indicate a month in which the facility had low stock of RDTs, with less than one month's estimated required RDTs (based on average from previous months' use) available in stock.

OSPSANTE data are available monthly for each public health facility but are not available for private facilities. No data are available to describe the specific day or week when RDT stock reached zero, or when additional stock was received. Months without a black horizontal line indicate that the facility had RDTs in stock

Interpretation

The only site which reported a stockout of RDTs (public urban site 1) showed consistent low used of RDT throughout the study period, with the majority of participants tested using microscopy. There was a small increase in the number of participants not tested in September, but the majority continued to be tested by microscopy. No stockouts were reported from public urban site 2, where participants received a mixture of RDT, microscopy and no testing throughout the study period.

At public rural site 1, logistics management data indicate low stock of RDTs in July and September, but no stockouts. However, very few participants received any confirmatory testing, with the exception of the first week of the study in early July. It is possible that low stock of RDTs contributed to low testing rates at this facility, but it is unexpected that there would be a complete lack of RDT stock unrecorded by OSPSANTE that led to almost no participants being tested.

At public rural site 2, the proportion of participants tested by RDT began high in the first week of July, before dropping as increasing numbers were not tested. The same pattern was seen in September. In July and October there was evidence of low stock of RDTs, but there was no report of low stock or stockout of RDTs in September. While low stock of RDTs could have led to reduced testing and a desire by staff to 'ration' their RDTs, this does not explain the low testing in September, when there appeared to be adequate stock of RDTs.
