# Supplementary material for: Management of uncomplicated malaria among children under five years at public and private sector facilities in Mali
Source: BMC Public Health. 2020 Dec 9;20:1888. doi: 10.1186/s12889-020-09873-1 (PMC7724888; doi:10.1186/s12889-020-09873-1)
Supplement: Supplementary file 3 — Additional file 3. Description of case management practices at all sites and correct/incorrect case management designation. [file 12889_2020_9873_MOESM3_ESM.pdf]

Supplementary File 3

Fomba et al. Management of uncomplicated malaria among children under five years at public and private sector facilities in Mali

Description of case management practices at all sites and correct/incorrect case management designation

	Parasitological testing			Treatment provided		
	**Scenario**	**n**	**%**	**Scenario**	**n**	**%**
ALL RECRUITING SITES	Tested by RDT	507	31.7	Negative test result, given no antimalarial drug of any type	120	7.5
	Tested by microscopy	287	17.9	Positive test result, given ACT and no other antimalarial	259	16.2
	Correctly managed	***794***	***49.6***	***Correctly managed***	***379***	***23.7***
	Not tested	807	50.4	Positive test result, given ACT and non-ACT antimalarial	106	6.6
				Positive test result, given non-ACT antimalarial but not ACT	185	11.5
				Positive test result, not given any type of antimalarial	23	1.4
				Negative test result, given ACT and no other antimalarial	66	4.1
				Negative test result, given ACT and non-ACT antimalarial	14	0.9
				Negative test result, given non-ACT antimalarial but not ACT	15	0.9
				Not tested, given ACT and no other antimalarial	197	12.3
				Not tested, given ACT and non-ACT antimalarial	142	8.9
				Not tested, given non-ACT antimalarial but not ACT	271	16.9
				Not tested, no antimalarial	197	12.3
				Tested, did not know result and given ACT and no other antimalarial	3	0.2
				Tested, result unknown, given ACT and non-ACT	0	0.0
				Tested, result unknown, given non-ACT but not ACT	2	0.1
				Tested, did not know result, no antimalarial	2	0.1
	Incorrectly managed	***807***	***50.4***	***Incorrectly managed***	***1223***	***76.3***
