# Supplementary material for: Management of uncomplicated malaria among children under five years at public and private sector facilities in Mali
Source: BMC Public Health. 2020 Dec 9;20:1888. doi: 10.1186/s12889-020-09873-1 (PMC7724888; doi:10.1186/s12889-020-09873-1)

Supplementary File 4

Fomba et al. Management of uncomplicated malaria among children under five years at public and private sector facilities in Mali

Proportion of children receiving specific drugs at public urban facilities

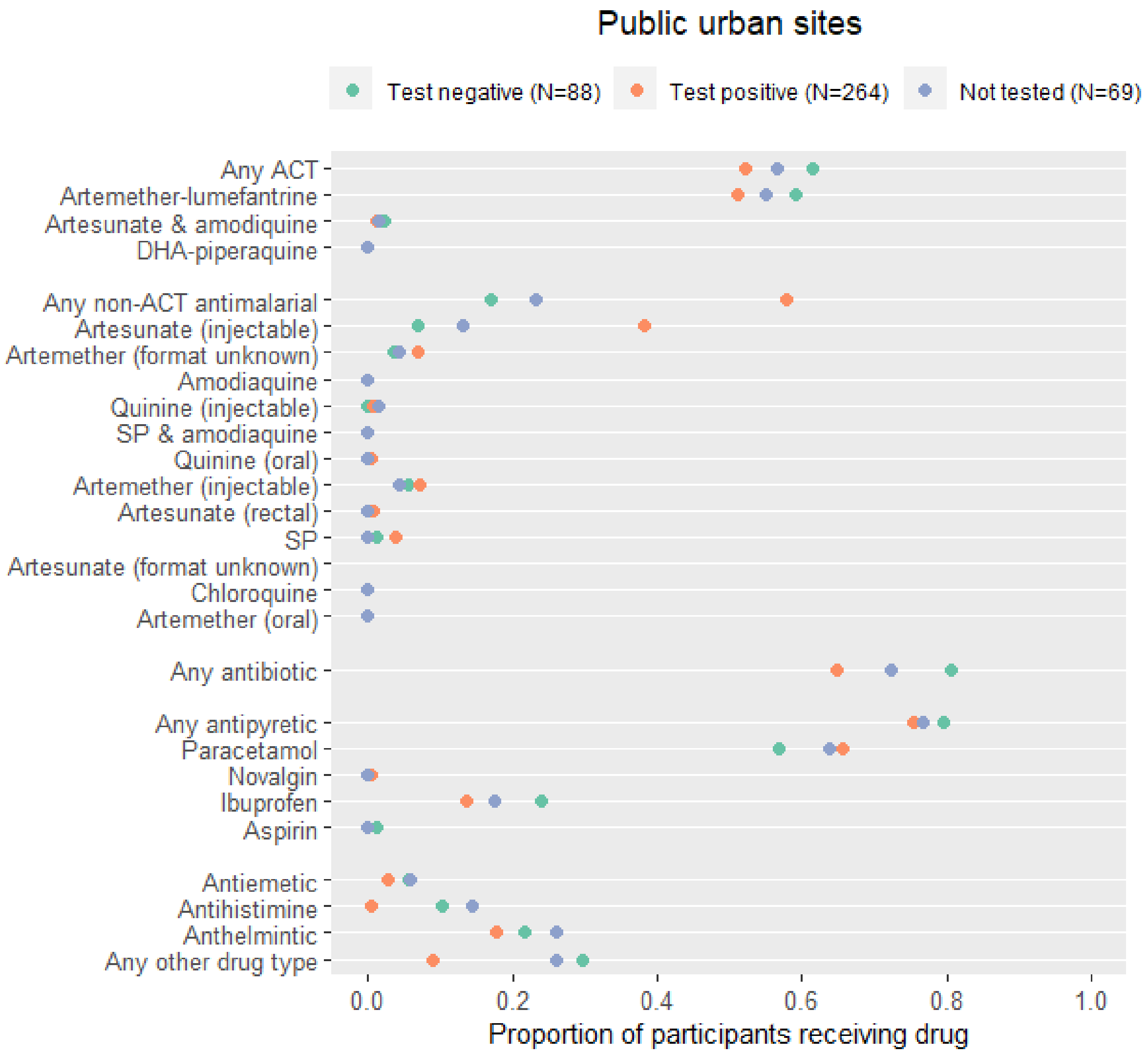

Proportion of children receiving specific drugs at public rural facilities

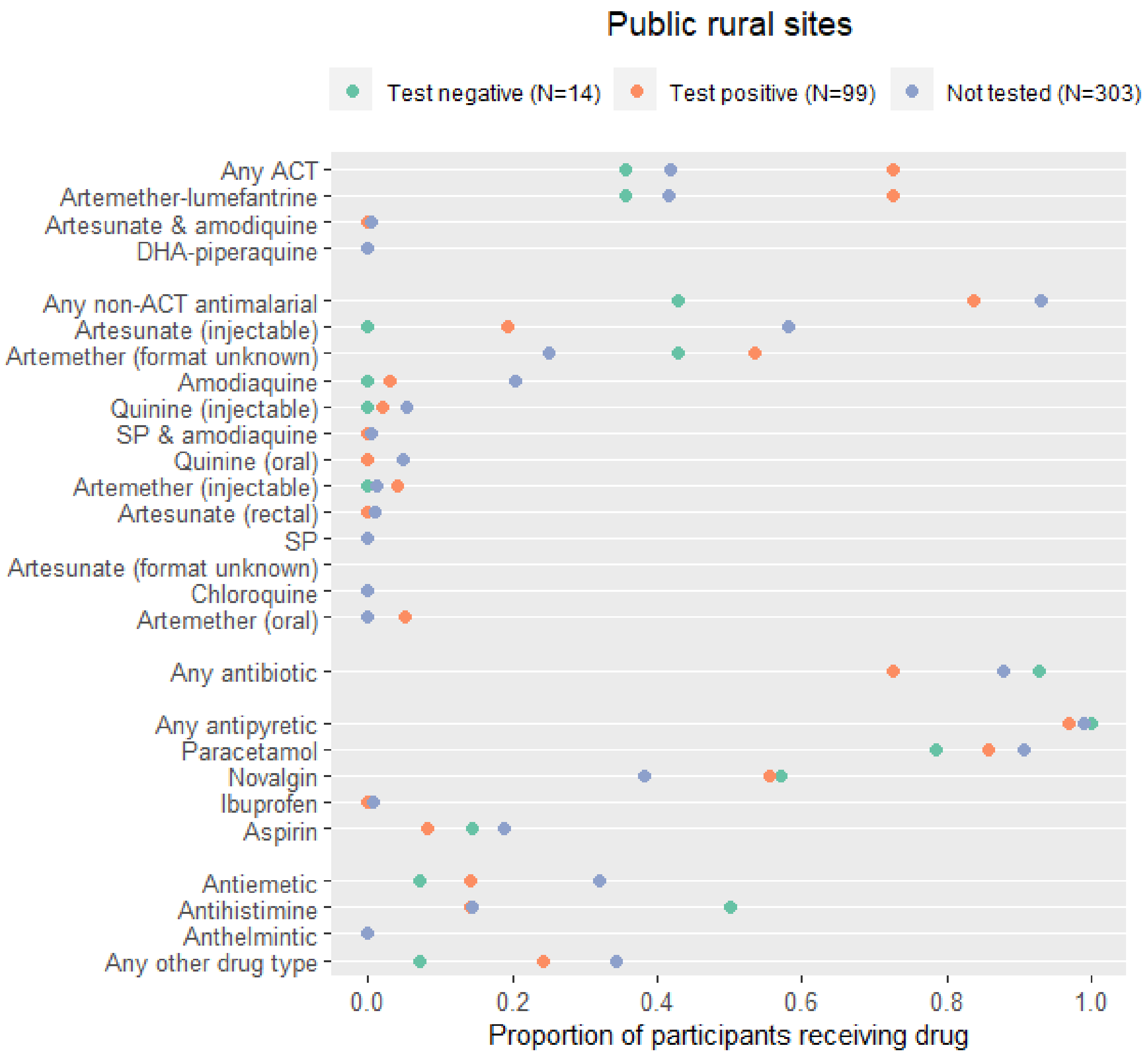

Proportion of children receiving specific drugs at community health worker sites

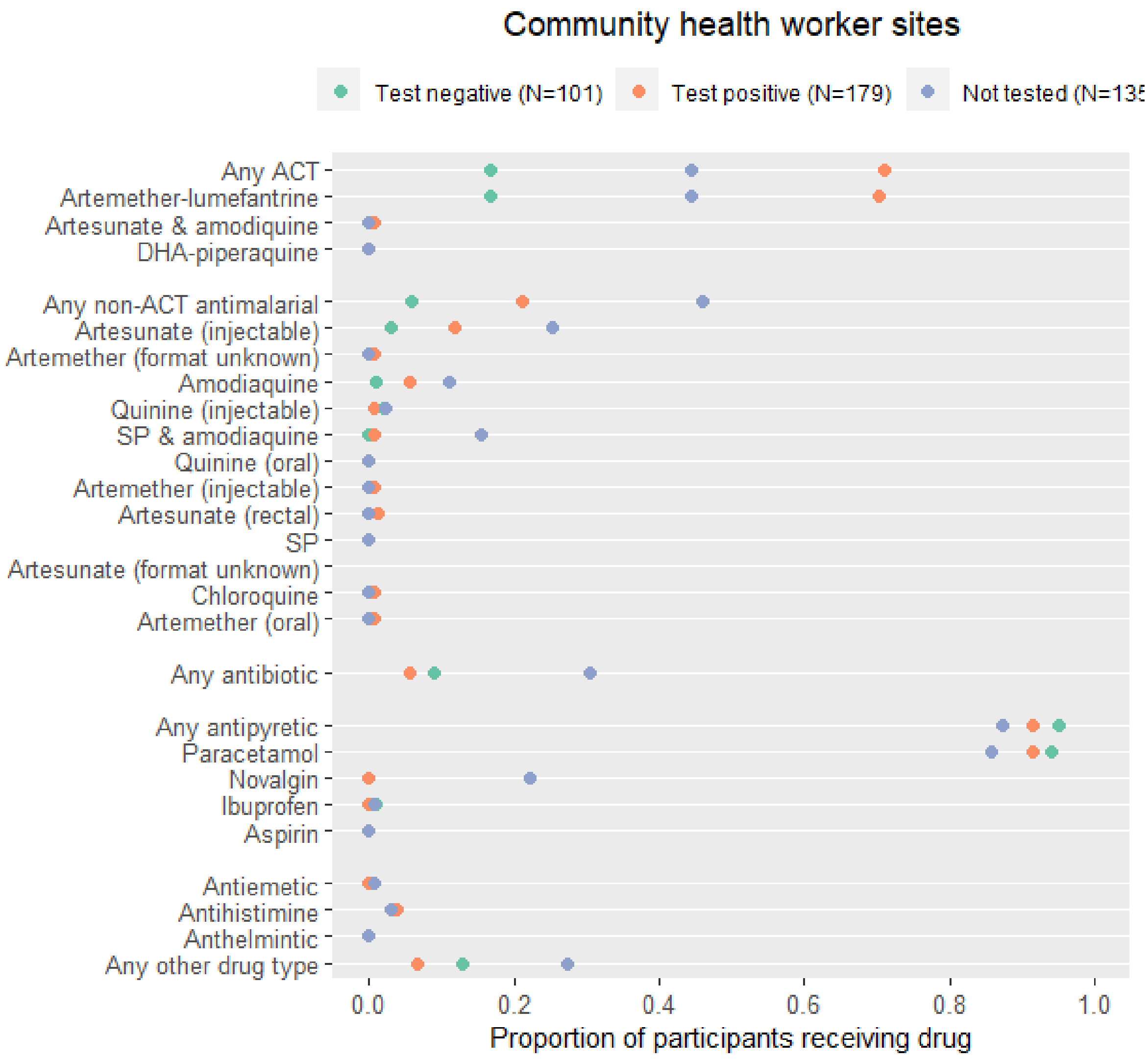

Proportion of children receiving specific drugs at private urban facilities

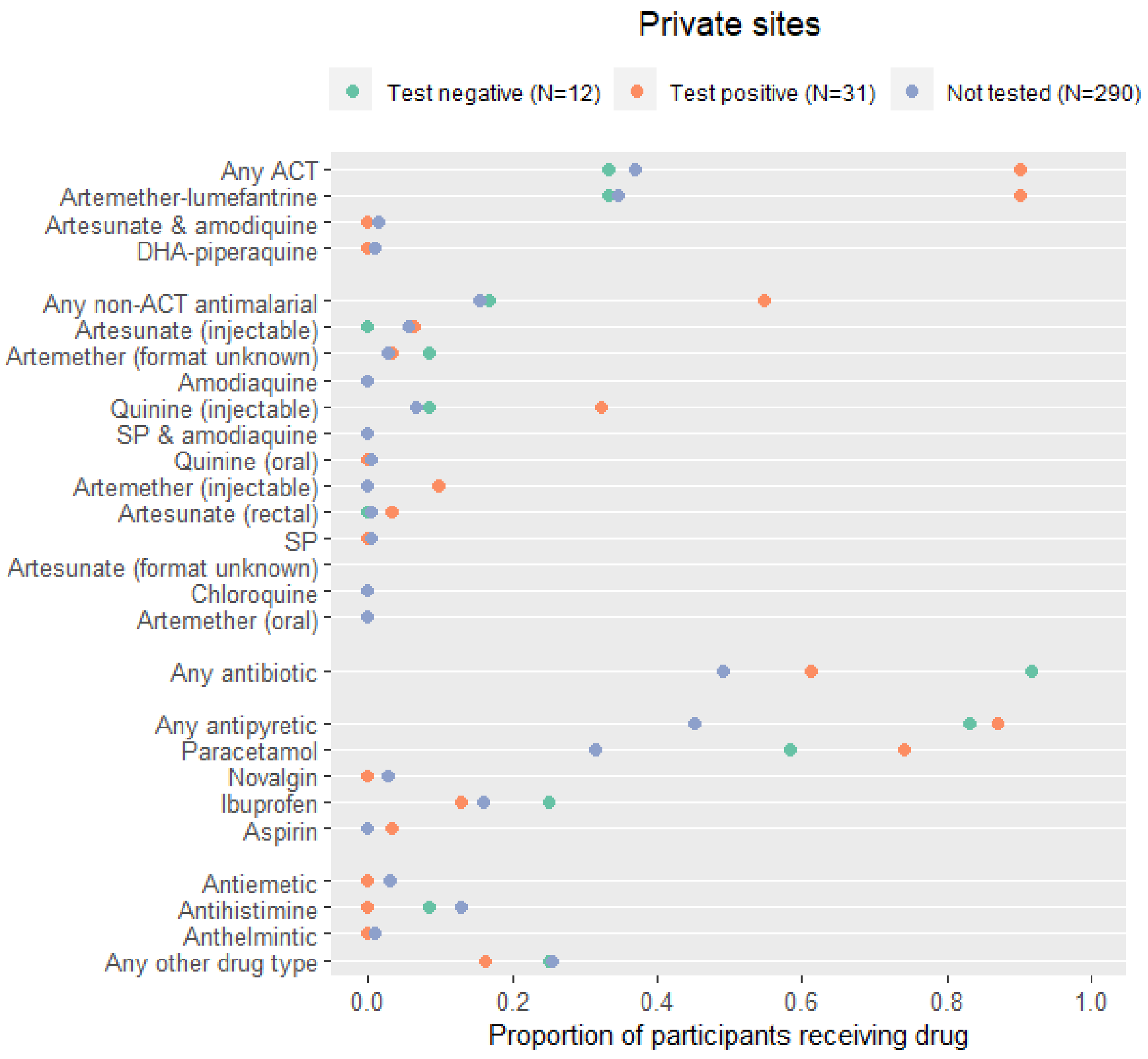

Supplement: Supplementary file 4 — Additional file 4. Proportion of children receiving specific drugs, described by each recruiting facility type. [file 12889_2020_9873_MOESM4_ESM.pdf]
