# Supplementary material for: Management of uncomplicated malaria among children under five years at public and private sector facilities in Mali
Source: BMC Public Health. 2020 Dec 9;20:1888. doi: 10.1186/s12889-020-09873-1 (PMC7724888; doi:10.1186/s12889-020-09873-1)
Supplement: Supplementary file 5 — Additional file 5. Summary of antimalarial drug prescription at public facility sites by study week, and corresponding OPSANTE data describing periods of ACT stockout. [file 12889_2020_9873_MOESM5_ESM.pdf]

Supplementary File 5

Fomba et al. Management of uncomplicated malaria among children under five years at public and private sector facilities in Mali

Weekly proportion of participants receiving ACT only, ACT and non-ACT, non-ACT only, or no antimalarials of any type at public urban and public rural sites, with indication of when stockouts of Artemether-Lumefantrine (AL) 6x1 dose (gray horizontal markers) and AL 6x2 dose (black horizontal markers) likely occurred, according to OSPSANTE data

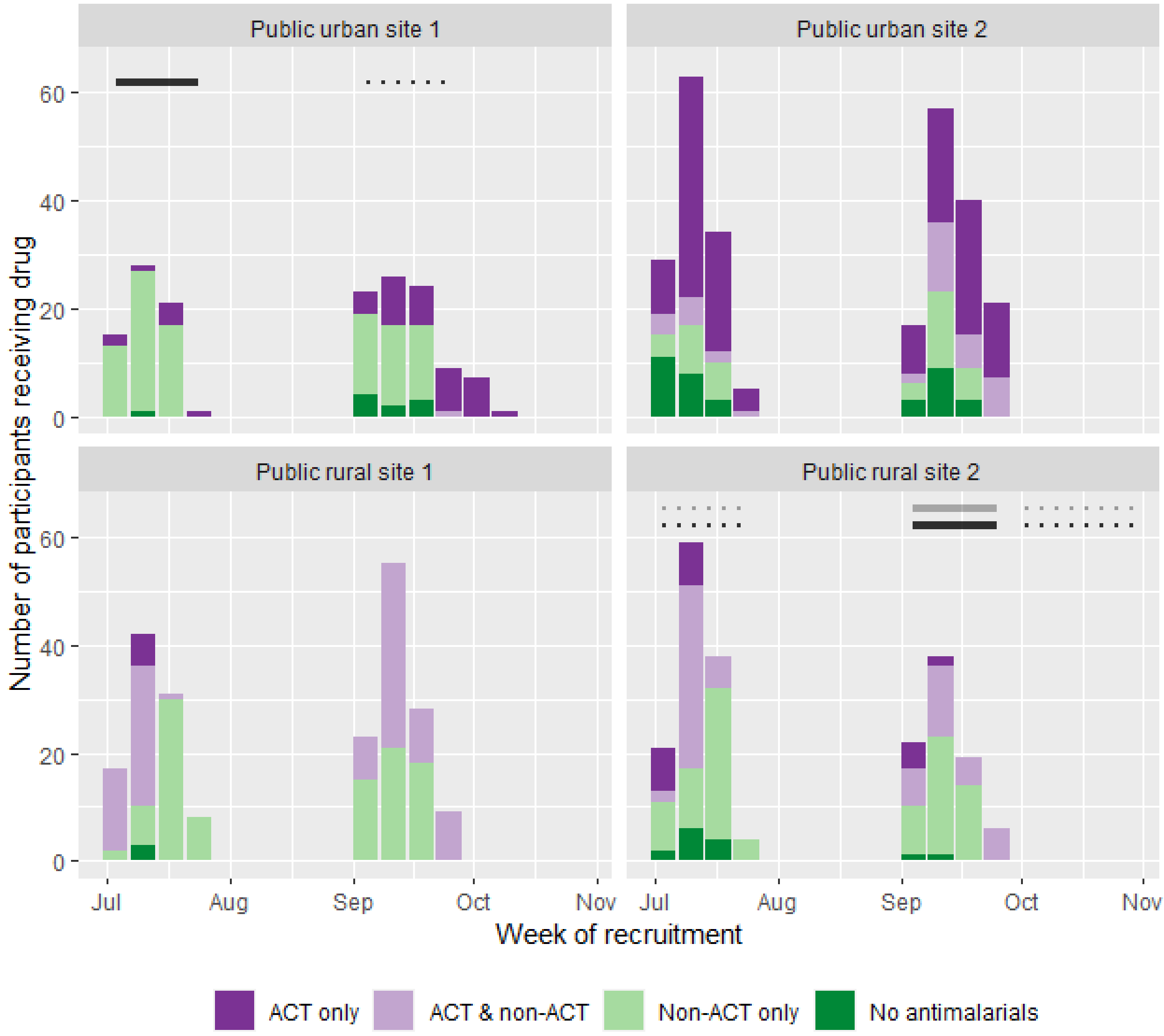

Data used

The above figure combines data collected as part of the study with routinely collected data in the Health Commodity Dashboard system used in Mali (OSPSANTE). Study data collected from the two public urban facilities and two public rural facilities participating in the study describe the weekly proportion children receiving ACT only, ACT plus a non-ACT, a non-ACT only, or no antimalarial of any type.

Solid gray horizontal lines indicate a month in which the OSPSANTE system recorded that the facility had no AL 6x1 packages in stock, while dotted grey horizontal lines indicate a month in which the facility had a low stock AL 6x1, with less than one month's estimated required AL 6x1 (based on average from previous months' use) available in stock. Solid black lines indicate stockouts of AL 6x2 packages, with dotted black lines indicating low stock of AL 6x2. OSPSANTE data are available monthly for each public health facility but are not available for private facilities. No data are available to describe the specific day or week when stock reached zero, or when additional stock was received. Months without a horizontal line indicate that the facility had both AL 6x1 and 6x2 in stock, while months with both gray and black horizontal lines indicate a stockout of both doses of AL.

Interpretation

There was evidence of stockout of one type of AL at public urban site 1 during the study, and stockout of both AL types at public rural site 2. It is possible that some instances of prescribing non-ACTs in these facilities were due to stockouts of ACT. However, there was no evidence of ACT stockout at the other two public facilities, both of which prescribed non-ACTs to participating children throughout the study period.
