# Supplementary material for: Management of uncomplicated malaria among children under five years at public and private sector facilities in Mali
Source: BMC Public Health. 2020 Dec 9;20:1888. doi: 10.1186/s12889-020-09873-1 (PMC7724888; doi:10.1186/s12889-020-09873-1)
Supplement: Supplementary file 6 — Additional file 6. Description of case management practices by study site type and correct/incorrect case management designation. [file 12889_2020_9873_MOESM6_ESM.pdf]

Supplementary File 6

Fomba et al. Management of uncomplicated malaria among children under five years at public and private sector facilities in Mali

Description of case management practices by study site type and correct/incorrect case management designation

	Parasitological testing			Treatment provided		
	**Scenario**	**n**	**%**	**Scenario**	**n**	**%**
**PUBLIC URBAN FACLITIES**	Tested by RDT	101	24.0	Negative test result, given no antimalarial drug of any type	26	6.2
	Tested by microscopy	251	59.6	Positive test result, given ACT and no other antimalarial	108	25.7
	Correctly managed	***352***	***83.6***	***Correctly managed***	***134***	***31.8***
	Not tested	69	16.4	Positive test result, given ACT and non-ACT antimalarial	30	7.1
				Positive test result, given non-ACT antimalarial but not ACT	123	29.2
				Positive test result, not given any type of antimalarial	3	0.7
				Negative test result, given ACT and no other antimalarial	47	11.2
				Negative test result, given ACT and non-ACT antimalarial	7	1.7
				Negative test result, given non-ACT antimalarial but not ACT	8	1.9
				Not tested, given ACT and no other antimalarial	35	8.3
				Not tested, given ACT and non-ACT antimalarial	4	1.0
				Not tested, given non-ACT antimalarial but not ACT	12	2.9
				Not tested, no antimalarial	18	4.3
				Tested, did not know result and given ACT and no other antimalarial	0	0.0
				Tested, result unknown, given ACT and non-ACT	0	0.0
				Tested, result unknown, given non-ACT but not ACT	0	0.0
				Tested, did not know result, no antimalarial	0	0.0
	Incorrectly managed	***69***	***16.4***	***Incorrectly managed***	***287***	***68.2***

	Parasitological testing			Treatment provided		
	Scenario	n	%	Scenario	n	%
PUBLIC RURAL FACLITIES	Tested by RDT	112	26.7	Negative test result, given no antimalarial drug of any type	7	1.7
	Tested by microscopy	1	0.2	Positive test result, given ACT and no other antimalarial	15	3.6
	Correctly managed	***113***	***26.9***	***Correctly managed***	***22***	***5.2***
	Not tested	307	73.1	Positive test result, given ACT and non-ACT antimalarial	57	13.6
				Positive test result, given non-ACT antimalarial but not ACT	26	6.2
				Positive test result, not given any type of antimalarial	1	0.2
				Negative test result, given ACT and no other antimalarial	1	0.2
				Negative test result, given ACT and non-ACT antimalarial	4	1.0
				Negative test result, given non-ACT antimalarial but not ACT	2	0.5
				Not tested, given ACT and no other antimalarial	13	3.1
				Not tested, given ACT and non-ACT antimalarial	115	27.4
				Not tested, given non-ACT antimalarial but not ACT	170	40.5
				Not tested, no antimalarial	9	2.1
				Tested, did not know result and given ACT and no other antimalarial	0	0.0
				Tested, result unknown, given ACT and non-ACT	0	0.0
				Tested, result unknown, given non-ACT but not ACT	0	0.0
				Tested, did not know result, no antimalarial	0	0.0
	Incorrectly managed	***307***	***73.1***	***Incorrectly managed***	***398***	***94.8***

	Parasitological testing			Treatment provided		
	Scenario	n	%	Scenario	n	%
COMMUNITY HEALTH WORKERS	Tested by RDT	283	66.6	Negative test result, given no antimalarial drug of any type	79	19.0
	Tested by microscopy	0	0.0	Positive test result, given ACT and no other antimalarial	112	27.0
	Correctly managed	***283***	***66.6***	***Correctly managed***	***191***	***46.0***
	Not tested	142	33.4	Positive test result, given ACT and non-ACT antimalarial	5	1.2
				Positive test result, given non-ACT antimalarial but not ACT	33	8.0
				Positive test result, not given any type of antimalarial	19	4.6
				Negative test result, given ACT and no other antimalarial	16	3.9
				Negative test result, given ACT and non-ACT antimalarial	1	0.2
				Negative test result, given non-ACT antimalarial but not ACT	5	1.2
				Not tested, given ACT and no other antimalarial	45	10.8
				Not tested, given ACT and non-ACT antimalarial	21	5.1
				Not tested, given non-ACT antimalarial but not ACT	46	11.1
				Not tested, no antimalarial	30	7.2
				Tested, did not know result and given ACT and no other antimalarial	2	0.5
				Tested, result unknown, given ACT and non-ACT	0	0.0
				Tested, result unknown, given non-ACT but not ACT	0	0.0
				Tested, did not know result, no antimalarial	1	0.2
	Incorrectly managed	***142***	***33.4***	***Incorrectly managed***	***224***	***54.0***

	Parasitological testing			Treatment provided		
	Scenario	n	%	Scenario	n	%
PRIVATE URBAN FACILITIES	Tested by RDT	11	3.3	Negative test result, given no antimalarial drug of any type	8	2.4
	Tested by microscopy	35	10.4	Positive test result, given ACT and no other antimalarial	14	4.2
	Correctly managed	***46***	***13.7***	***Correctly managed***	***22***	***6.5***
	Not tested	290	86.3	Positive test result, given ACT and non-ACT antimalarial	14	4.2
				Positive test result, given non-ACT antimalarial but not ACT	3	0.9
				Positive test result, not given any type of antimalarial	0	0.0
				Negative test result, given ACT and no other antimalarial	2	0.6
				Negative test result, given ACT and non-ACT antimalarial	2	0.6
				Negative test result, given non-ACT antimalarial but not ACT	0	0.0
				Not tested, given ACT and no other antimalarial	105	31.3
				Not tested, given ACT and non-ACT antimalarial	2	0.6
				Not tested, given non-ACT antimalarial but not ACT	43	12.8
				Not tested, no antimalarial	140	41.7
				Tested, did not know result and given ACT and no other antimalarial	1	0.3
				Tested, result unknown, given ACT and non-ACT	0	0.0
				Tested, result unknown, given non-ACT but not ACT	1	0.3
				Tested, did not know result, no antimalarial	1	0.3
	Incorrectly managed	***290***	***86.3***	***Incorrectly managed***	***314***	***93.5***
